# The Potential Role of *Moringa oleifera* Lam. Leaf Proteins in Moringa Allergy by Functionally Activating Murine Bone Marrow-Derived Dendritic Cells and Inducing Their Differentiation toward a Th2-Polarizing Phenotype

**DOI:** 10.3390/nu16010007

**Published:** 2023-12-19

**Authors:** Chuyu Xi, Wenjie Li, Xiaoxue Liu, Jing Xie, Shijun Li, Yang Tian, Shuang Song

**Affiliations:** 1 College of Food Science and Technology, Yunnan Agricultural University, Kunming 650201, China; xichuyu@stu.ynau.edu.cn (C.X.); liwenjie@stu.ynau.edu.cn (W.L.); liuxiaoxue@stu.ynau.edu.cn (X.L.); jingxie0624@163.com (J.X.); lsj7783@126.com (S.L.); tianyang@ynau.edu.cn (Y.T.); 2 Yunnan Key Laboratory of Precision Nutrition and Personalized Food Manufacturing, Yunnan Agricultural University, Kunming 650201, China; 3 Engineering Research Center of Development and Utilization of Food and Drug Homologous Resources, Ministry of Education, Yunnan Agricultural University, Kunming 650201, China

**Keywords:** *Moringa oleifera* Lam., *M. oleifera* leaf proteins, food allergy, dendritic cells, Th2-promoting profile

## Abstract

*Moringa oleifera* leaves are an inexpensive substitute for staple foods. Despite limited data, *Moringa oleifera* leaf protein (Mo-Pr) may be allergenic in BALB/c mice. In mouse models and allergic patients, dendritic cells (DCs) may be involved in food allergy. In addition, some allergens, including food allergens, can directly activate DCs and induce Th2 polarization. We investigated whether Mo-Pr can modulate the functional profile of murine bone marrow-derived dendritic cells (BMDCs) in vitro. BMDCs were obtained from mouse bone marrow cultured with granulocyte–macrophage colony-stimulating factor (GM-CSF) for 7 days and then treated with lipopolysaccharide (LPS) or Mo-Pr. BMDC phenotypes were evaluated via flow cytometry, cytokine production was assessed using ELISA, the expression of key genes was studied using qRT-PCR, the effects on T-cell differentiation were investigated using mixed lymphocyte reaction (MLR), and transcriptional changes in BMDCs were investigated using RNA-Seq. Mo-Pr-specific IgE was investigated in recipient serum after BMDC transfer. Mo-Pr treatment significantly induced BMDC maturation, increased the expression of CD80/86 and MHC II, resulted in the production of IL-12 and TNF-α, and induced T-cell differentiation. Mo-Pr treatment stimulated BMDCs’ expression of the Th2 promoters OX40L and TIM-4, induced the production of the Th2-type chemokines CCL22 and CCL17, and decreased the Th1/Th2 ratio in vitro. Healthy recipients of Mo-Pr-treated BMDCs produced Mo-Pr-specific IgE.

## 1. Introduction

*Moringa oleifera* (*M. oleifera*), a member of the *Moringaceae* family, is a perennial tropical deciduous tree native to the Himalayan region of India, Pakistan, Bangladesh, and Afghanistan [1]. All parts of the *M. oleifera* plant are edible, but the leaves are generally the most commonly used part [2] because of their effectiveness in combating malnutrition [3]. The leaves are rich in protein and also provide an important source of minerals, vitamins, beta-carotene, amino acids, and phenolics, nutrients that may not be easily available to populations in underdeveloped or developing countries [4]. It is important to note that some individuals may be allergic to the consumption of *M. oleifera* leaves. A case of occupational asthma due to *M. oleifera* seed powder in the workplace of a worker in a cosmetics manufacturer has been reported [5]. There have been several studies on *M. oleifera* leaf allergens. Jie Zhang et al. successfully sensitized BALB/c mice to *M. oleifera* leaf protein through oral administration [6]. Giovanni D’Auria et al. identified potential *M. oleifera* leaf allergens, including morintides and nsLTP, through proteomic analysis [7]. Janitha Iddagoda et al. determined the molecular weights of the *M. oleifera* leaf allergens to be 14, 23, 35, 43, and 48 kDa using Western blotting with serum from patients with *M. oleifera* leaf allergy [8]. While the current evidence suggests the existence of potential allergens in *M. oleifera* leaf, the mechanism of allergy caused by *M. oleifera* leaf protein or *M. oleifera* leaf allergen remains unclear.

Food allergy is a group of diseases caused by an adverse immune response to food antigens. Food-allergic diseases with acute onset of symptoms after ingestion are usually mediated by allergen-specific IgE [9]. Dendritic cells (DCs) are the primary antigen-presenting cells (APCs) of the immune system [10]. They drive the differentiation of antigen-specific CD4^+^ Th2 cells, which requires active induction by various molecules [11]. After exposure to food antigens, DCs mature progressively. This enhances their capacity to present antigens, increases the expression of related surface costimulatory molecules, and induces the differentiation of naive T cells through the secretion of cytokines [12]. Our previous studies conducted in our laboratory have shown an increased proportion of Th2 cells in the spleens of mice that were sensitized to *M. oleifera* leaf protein (Mo-Pr) (data described in another manuscript). According to our research, Mo-Pr has the potential to induce DC polarization, resulting in the differentiation of CD4^+^ T cells into Th2 cells. This process may lead to *M. oleifera* leaf allergy.

This study involves an investigation of the effects of Mo-Pr on DCs, specifically its ability to induce maturation and Th2 polarization of Mo-Pr-stimulated DCs. DCs can be categorized into two functional states: immature and mature. Only mature DCs are capable of initiating an immune response. DC maturation is characterized by an increase in surface costimulatory molecules, such as CD80/86, MHC II, and cytokines, as well as an enhanced ability to stimulate T-cell differentiation [13]. In this study, we cocultured Mo-Pr with mouse bone marrow-derived dendritic cells (BMDCs) in vitro. Our hypothesis was verified through flow cytometry, ELISA, and mixed lymphocyte reaction (MLR). The increase in Th2 cell differentiation is believed to be due to the polarization of DCs. These ideas are based on studies conducted with the peanut allergen Ara h 1, which can directly activate DCs and induce Th2 polarization in vitro [14]. This study aims to determine if Mo-Pr has similar capabilities as Ara h 1. Specifically, the study focuses on the surface costimulatory molecules OX40L and TIM-4, which are believed to be associated with DC Th2 polarization. It is essential for DCs to express OX40L to induce optimal primary and memory Th2 responses [15]. One study demonstrated that oral cholera toxin (CT) induces DC maturation and increases OX40L in mesenteric lymph node (MLN) DCs [16]. Additionally, the expression of TIM-4 on DCs and its binding to TIM-1 on T cells has been shown to impact Th2 cell development [17]. In vivo, exposure to ovalbumin (OVA) as an antigen and staphylococcal enterotoxin B (SEB) as an adjuvant resulted in OVA-specific Th2 differentiation and intestinal allergic responses. The blockade of TIM-4 or its ligand, TIM-1, reduced Th2 differentiation and intestinal allergy [18]. Similar results were observed when peanut was the antigen, and CT was the adjuvant [19]. We hypothesize that the expression level of DC surface molecules can be used to evaluate the maturation of DCs, and some of these molecules are specific to the Th2 polarization. Our hypothesis was tested through a series of experiments.

## 2. Materials and Methods

### 2.1. BALB/c Mice

Hunan Slakejing Da Laboratory Animal Co., Ltd. (Changsha, China) provided male BALB/c and C57 mice (5–6 weeks old, SPF) maintained on an allergen-free diet under pathogen-free conditions. These mice were maintained in an animal room with a temperature of 23 ± 3 °C and humidity of 50 ± 10%, and an alternating 12:12 h light–dark cycle, all under SPF conditions. This study was conducted in accordance with the Declaration of Helsinki and approved by the Ethical Committee of Experimental Animal Care of Yunnan Agricultural University (Protocol Code: 202107013 and Date of Approval: 19 July 2021).

### 2.2. Extraction of M. oleifera Leaf Protein

Fresh *M. oleifera* leaves were homogenized in PBS at a material-to-liquid ratio of 1:6 and immersed at 4 °C for 4 h with stirring at 1 h intervals. Centrifugation at 3500 rpm for 20 min yielded a filtrate from which the supernatant was collected. The precipitates were incubated with 70% ammonium sulfate overnight and then spun again to harvest the pellet. The pellet was then solubilized with PBS, dialyzed for 2–4 days, and finally lyophilized under vacuum. The collected product was the *M. oleifera* leaf protein (Mo-Pr). Mo-Pr was stored at −20 °C.

### 2.3. BMDC Generation and Differentiation In Vitro

BMDCs were generated from the bone marrow cells (BMCs) of BALB/c mice as previously described [20], and BMCs were obtained from the femur and tibia of male mice. Then, 10^7^/mL cells were transferred to a 24-well cell culture plate (LABSELECT, Beijing Labgic Technology Co., Ltd., Beijing, China) filled with 1 mL of an enriched RPMI 1640 medium (Biosharp, Beijing Labgic Technology Co., Ltd., Beijing, China) containing 10% fetal bovine serum (Biological Industries, Biological Industries Israel Beit Haemek, Ltd., Kibbutz Beit-Haemek, Israel), 2% penicillin–streptomycin liquid (100×) (Beijing Solarbio Science and Technology Co., Ltd., Beijing, China), and 10 ng/mL granulocyte–macrophage colony-stimulating factor (GM-CSF) (PeproTech, Cranbury, NJ, USA). After 7 days of culture, floating BMDCs were collected.

### 2.4. Flow Cytometry

BMDCs were treated with Mo-Pr (50, 100, or 200 μg/mL), lipopolysaccharide (LPS) (1 μg/mL) (Biosharp, Beijing Labgic Technology Co., Ltd., Beijing, China), or RPMI 1640 for 48 h. The BMDCs were washed twice and resuspended in a flow cytometry staining buffer (MultiSciences Biotech Co., Ltd., Hangzhou, China). TruStain FcXTM PLUS (anti-mouse CD16/32) antibody (BioLegend, San Diego, CA, USA) was added to the culture of BMDCs at 4 °C for 10 min, followed by three washes with the flow cytometry staining buffer. PE anti-CD11c, PE anti-CD80, PE anti-CD86, APC anti-MHC class II (I-A/I-E), APC anti-OX40L (CD252) (Proteintech Group, Inc., 5500 Pearl Street, Suite 400., Rosemont, IL, USA), and Alexa Fluor^®^ 674 anti-TIM-4 antibodies (BioLegend, San Diego, CA, USA) were added to the culture of BMDCs at 4 °C for 20 min. After filtration through 200 mesh nylon, the BMDCs were analyzed using a BD Accuri C6 flow cytometer (BD Biosciences, Accuri Cytometers Inc., 173 Parkland Plaza, Ann Arbor, MI, USA).

### 2.5. Measurement of Cytokines Using ELISA

BMDCs were treated with Mo-Pr (50, 100, or 200 μg/mL), LPS (1 μg/mL), or RPMI 1640 for 48 or 72 h. Cell supernatants were assayed for cytokines using commercial kits for TNF-α and IL-12p70 (NeoBioscience Technology Co., Ltd., Shenzhen, China) according to the manufacturer’s instructions.

### 2.6. qRT-PCR

BMDCs were treated with Mo-Pr (100 μg/mL), LPS (1 μg/mL), or RPMI 1640 for 48 h. Total RNA from BMDCs was isolated from 1 × 10^7^ cells using an RNA Easy Fast Tissue/Cell Kit (TIAGENBIOTECH (BEIJING) Co., Ltd., Beijing, China) according to the manufacturer’s instructions. Reverse transcription reactions were performed using a FastKing RT Kit (with gDNase) (TIAGENBIOTECH (BEIJING) Co., Ltd., Beijing, China) according to the manufacturer’s instructions. Quantitative real-time PCR was performed on a LightCycler 480 (Roche Diagnostics Deutschland GmbH, Sandhoferstrasse 116, Mannheim, Germany) using TB Green^®^ Premix Ex TaqTM II (Tli RNaseH Plus) (Takara Biomedical Technology (Beijing) Co., Ltd., Beijing, China) according to the manufacturer’s protocol. Primers and probes for mouse CCL17, CCL22, and GAPDH for TaqMan gene expression assays were purchased from Generay Biotech (Shanghai Generay Biotech Co., Ltd., Shanghai, China). The sequences of all primers in this study are shown in Table 1. Fold changes were calculated using the delta–delta cycle threshold (ΔΔCT) method.

### 2.7. Mixed Lymphocyte Reaction (MLR)

BMDCs were treated with Mo-Pr (100 μg/mL), LPS (1 μg/mL), or RPMI 1640 for 48 h. Lymphocytes (LYM) were generated from the spleens of C57 mice (5–6 weeks old, male). After the collected mouse spleen cells were cultured for 4 h, the collected floating cells were LYM. The BMDCs were then washed and cultured in a 24-well cell culture plate with freshly isolated allogeneic LYM (BMDCs/LYM ratio = 1/5) for 5 days. LYM were stained with CFSE (BD Biosciences, Accuri Cytometers Inc., 173 Parkland Plaza, Ann Arbor, MI, USA) on day 1 or with a mouse Th1/Th2 staining kit (MultiSciences Biotech Co., Ltd., Hangzhou, China) on day 7. Lymphocyte proliferation was determined via flow cytometry.

### 2.8. Inoculation of BMDCs

BMDCs were treated with Mo-Pr (100 μg/mL), or RPMI 1640 for 48 h. Fifteen BALB/c mice were divided into three groups (*n* = 5 each): normal saline (NS), RPMI 1640-treated BMDCs, and Mo-Pr-treated BMDCs. BMDCs were injected intraperitoneally into the recipient BALB/c mice (5 × 10^6^ cells per mouse) on day 0 and day 7. Mo-Pr (30 mg/kg b.w.) was administered intragastrically to the mice on day 14. At 24 h after gavage, abdominal blood was collected from mice under CO_2_ anesthesia. Serum Mo-Pr-specific IgE was measured by ELISA as described previously [21].

### 2.9. RNA-Seq

#### 2.9.1. RNA Extraction

BMDCs were treated with Mo-Pr (100 μg/mL), LPS (1 μg/mL), or RPMI 1640 for 4 h. Total RNA was extracted from the tissue using TRIzol^®^ reagent (Molecular Research Center, Inc., Cincinnati, OH, USA) according to the manufacturer’s instructions, and genomic DNA was eliminated using DNase I (Takara Biomedical Technology (Beijing) Co., Ltd., Beijing, China).

#### 2.9.2. Library Preparation and Sequencing

At Shanghai Majorbio Biopharm Biotechnology Co., Ltd. (Shanghai, China), RNA purification, reverse transcription, library construction, and sequencing were performed according to the manufacturer’s instructions (Illumina, San Diego, CA, USA).

The data have been uploaded to the NCBI database (BioProject: PRJNA1036700).

#### 2.9.3. Differential Expression Analysis and Functional Enrichment

The data were analyzed on the online platform of Majorbio Cloud Platform (www.majorbio.com, accessed on 1 March 2023). To identify the differentially expressed genes (DEGs) between two different groups, the expression level of each gene was determined according to the transcripts per million reads (TPM) method. In addition, functional enrichment analysis including GO (Gene Ontology, http://www.geneontology.org, accessed on 1 March 2023) and KEGG (Kyoto Encyclopedia of Genes and Genomes, http://www.genome.jp/kegg/, accessed on 1 March 2023) analysis was performed to determine which DEGs showed significant enrichment of GO terms and metabolic pathways at *p*-adjusted ≤ 0.05 compared with the whole-transcriptome background. GO functional enrichment and KEGG pathway analysis were carried out using Goatools (https://github.com/tanghaibao/Goatools, accessed on 1 March 2023) and KOBAS (http://kobas.cbi.pku.edu.cn/home.do, accessed on 1 March 2023).

### 2.10. Statistical Analysis

FlowJo v10.8.1 (BD Biosciences, Accuri Cytometers Inc., 173 Parkland Plaza, Ann Arbor, MI, USA.) was used for all statistical tests for flow cytometry. GraphPad Prism 8.0.2 (GraphPad Software Inc., San Diego, CA, USA) was used for all statistical tests. Statistical significance was determined using the nonparametric Kruskal–Wallis test for multiple comparisons with Dunn’s post-test for ANOVA or Student’s *t*-test. Statistical significance was defined as *p* < 0.05.

## 3. Results

### 3.1. Modulation of BMDC Maturation by Mo-Pr

**Figure 1:**
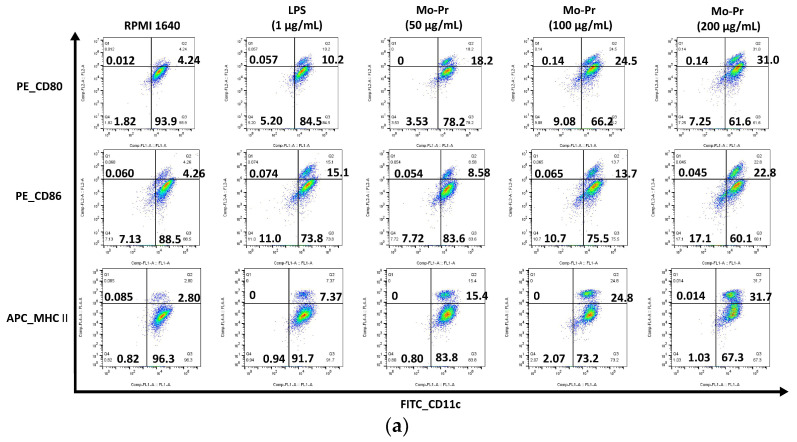

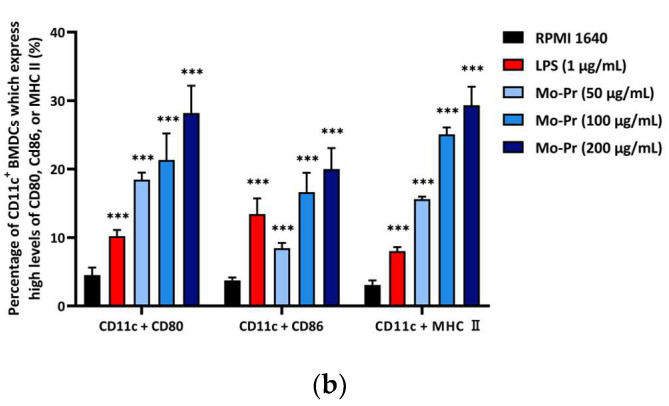
BMDCs (1 × 10^6^/mL) were cultured for 48 h with or without Mo-Pr (50, 100, or 200 μg/mL) or LPS (1 μg/mL), and the expression of CD11c, CD80, CD86, and MHC II was evaluated via flow cytometry. Representative histograms are shown in (**a**), while the mean ± SEM (*n* = 6) is shown in (**b**). *** *p* < 0.001 vs. controls.

**Figure 2:**
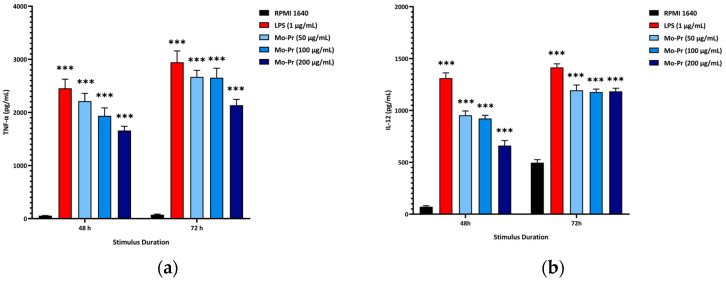
BMDCs (1 × 10^6^/mL) were cultured for 48 h or 72 h with or without Mo-Pr (50, 100, or 200 μg/mL) or LPS (1 μg/mL), and the production of IL-12 and TNF-α was measured by ELISA: (**a**) IL-12; (**b**) TNF-α. The mean ± SEM (*n* = 8) is shown in (**a**,**b**). *** *p* < 0.001 vs. controls.

**Figure 3:**
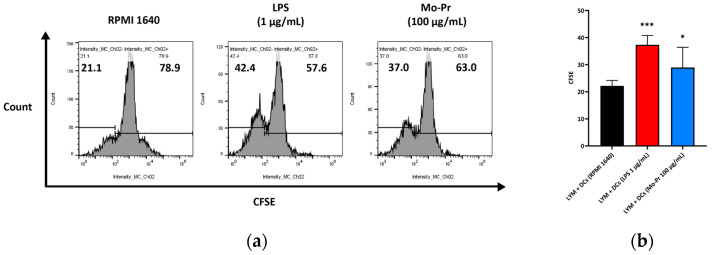
BMDCs (1 × 10^6^/mL) were cultured with or without Mo-Pr (100 μg/mL) or LPS (1 μg/mL) for 48 h and then cultured with C57 mouse splenic LYM (cell ratio, 1:5). LYM proliferation was assessed with CFSE staining. Representative histograms are shown in (**a**), while the mean ± SEM (*n* = 8) is shown in (**b**). * *p* < 0.05 and *** *p* < 0.001 vs. controls.

We first investigated whether Mo-Pr could promote the maturation of BMDCs in vitro. After Mo-Pr treatment, the expression of costimulatory molecules in BMDCs was increased. BMDCs treated with 50, 100, or 200 μg/mL Mo-Pr or 1 μg/mL LPS (positive control) for 48 h matured and showed an upregulated expression of CD80, CD86, and MHC II, as shown in Figure 1. In addition to the structural and phenotypic changes in BMDCs induced by Mo-Pr, these functionally mature BMDCs were also found to secrete increased levels of IL-12 and TNF-α, as shown in Figure 2. Interestingly, cytokine secretion gradually stabilized after 72 h. Mature BMDCs have a greater ability to stimulate LYM proliferation. We performed an allogeneic MLR to evaluate the maturation of Mo-Pr-treated BMDCs by assessing lymphoproliferative capacity. Compared with RPMI 1640-treated BMDCs, Mo-Pr-treated BMDCs had a greater ability to stimulate LYM proliferation, as shown in Figure 3.

### 3.2. Mo-Pr Polarizes BMDCs toward a Th2-Promoting Profile

**Figure 4:**
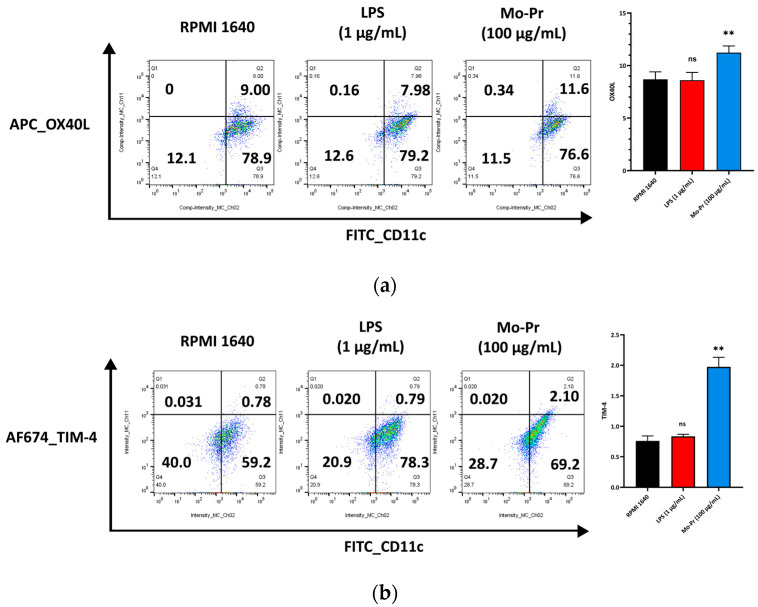
BMDCs (1 × 10^6^/mL) were cultured for 48 h with or without Mo-Pr (100 μg/mL) or LPS (1 μg/mL), and the expression of OX40L and TIM-4 was evaluated via flow cytometry: (**a**) OX40L; (**b**) TIM-4. In (**a**,**b**), representative histograms are shown in the left panels, while the mean ± SEM (*n* = 4) is shown in the right panel. ** *p* < 0.01 and ns *p* > 0.05 vs. controls.

**Figure 5:**
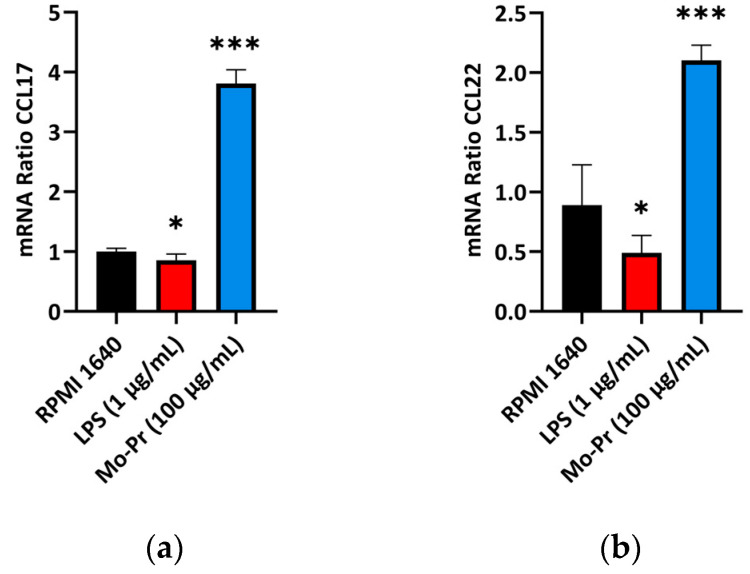
BMDCs (1 × 10^6^/mL) were cultured for 48 h with or without Mo-Pr (100 μg/mL) or LPS (1 μg/mL), and the production of CCL17 and CCL22 was measured using qRT-PCR: (**a**) CCL17; (**b**) CCL22. In (**a**,**b**), the mean ± SEM (*n* = 6) is shown. * *p* < 0.05 and *** *p* < 0.001 vs. controls.

**Figure 6:**
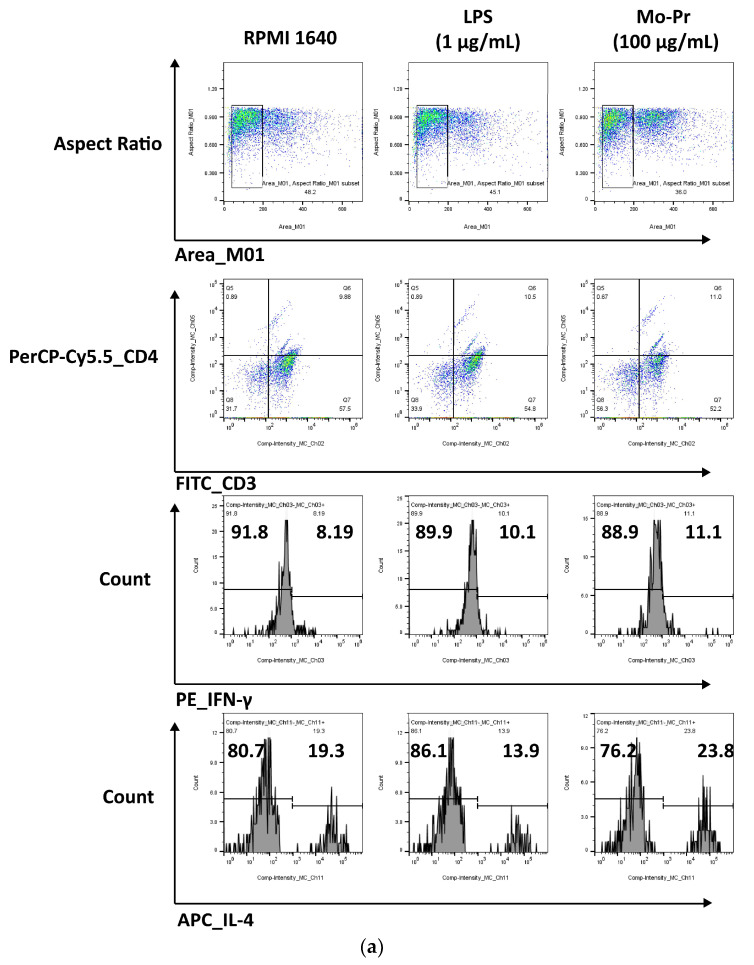

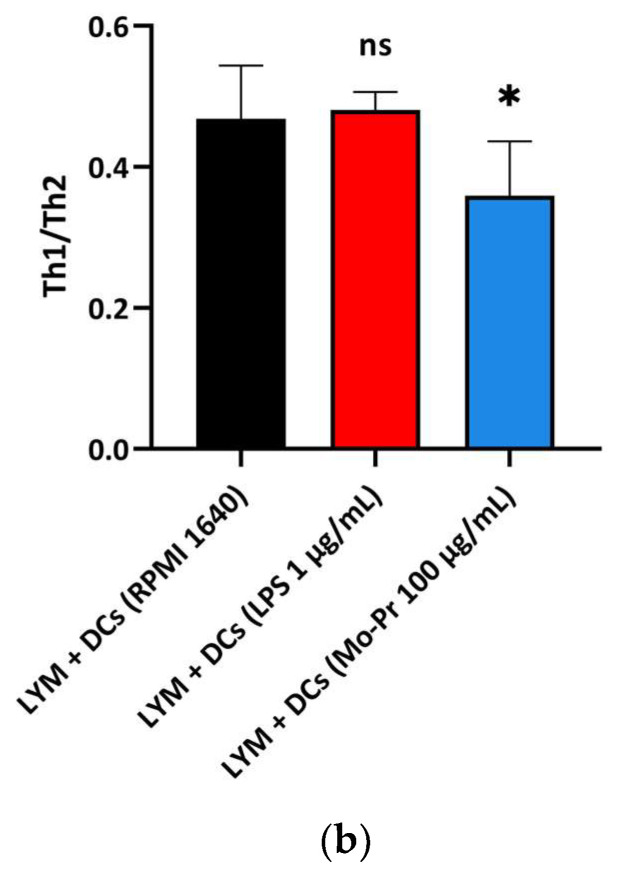
BMDCs (1 × 10^6^/mL) were cultured with or without Mo-Pr (100 μg/mL) or LPS (1 μg/mL) for 48 h and then cultured with C57 mouse splenic LYM (cell ratio, 1:5). The Th1/Th2 ratio was determined via flow cytometry to analyze the effect of LPS (1 μg/mL)-treated or Mo-Pr (100 μg/mL)-treated BMDCs on the stimulation of allogeneic lymphocyte differentiation. Representative histograms are shown in (**a**), while the mean ± SEM (*n* = 5) is shown in (**b**). * *p* < 0.05 and ns *p* > 0.05 vs. controls.

**Figure 7:**
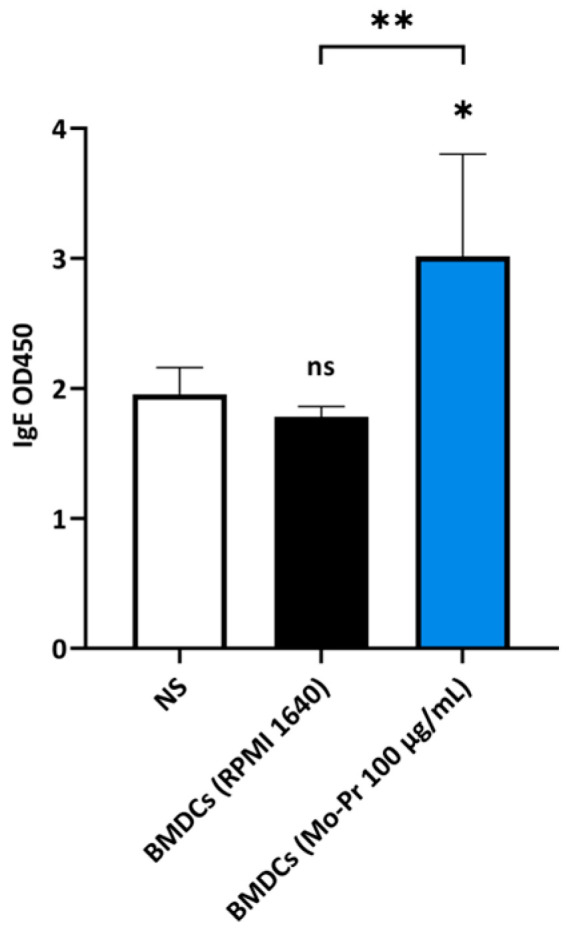
BALB/c mice were injected twice with Mo-Pr-treated BMDCs and orally gavaged once with Mo-Pr to evaluate Mo-Pr-specific IgE in the serum. The mean ± SEM (*n* = 5) is shown. * *p* < 0.05, ** *p* < 0.01 and ns *p* > 0.05 vs. controls.

OX40L and TIM-4 are two costimulatory molecules expressed on DCs that play an important role in allergic responses [11]. OX40–OX40L interactions modulate the differentiation and activity of CD4^+^ T cells [22]. For food allergens, Th2 polarization is mediated by OX40L [16]. TIM-4 expressed by mucosal DCs plays a critical role in food antigen-specific Th2 differentiation and intestinal allergy [18,23]. Considering the important role of OX40–OX40L interaction and TIM-4 in the induction of allergic inflammation, we investigated whether Mo-Pr could stimulate the expression of OX40L and TIM-4 in BMDCs. The culture of BMDCs with Mo-Pr (100 μg/mL) resulted in an increased expression of OX40L (Figure 4a) and TIM-4 (Figure 4b). It is generally believed that LPS polarizes DCs toward a Th1-promoting profile; the culture of BMDCs with LPS (1 μg/mL) resulted in a decreased expression of OX40L (Figure 4a), whereas TIM-4 expression was not affected (Figure 4b). Furthermore, the incubation of BMDCs with Mo-Pr also stimulated the production of CCL22 and CCL17 (Figure 5a,b), two chemokines capable of recruiting Th2 cells to the sites of allergic inflammation [24]. We performed allogeneic MLR. The results showed that allogeneic lymphocytes cocultured with Mo-Pr-stimulated BMDCs showed increased differentiation into Th2 cells (Figure 6). The production of antigen-specific IgE is one of the hallmarks of food allergy [25]. The inoculation of BMDCs demonstrated that the Mo-Pr stimulation of BMDCs in vitro can induce the production of Mo-Pr-specific IgE in healthy mice (Figure 7).

### 3.3. The MAPK Signaling Pathway May Play an Important Role in Mo-Pr-Mediated Induction of BMDC Th2 Polarization

**Figure 8:**
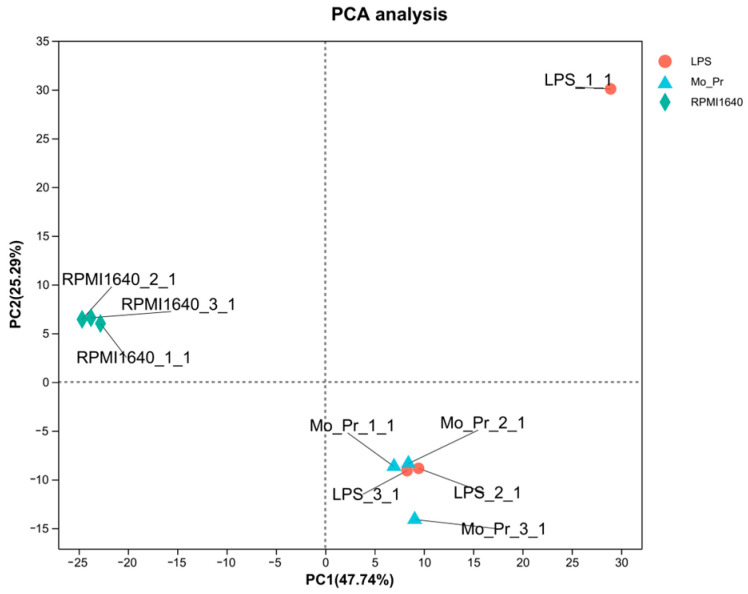
Principal component analysis (PCA) of the whole transcriptome of BMDCs in the LPS group, Mo-Pr group, and RPMI 1640 group. Different colors in the figure represent different groups, and shapes represent different samples (*n* = 3).

To determine why Mo-Pr or LPS stimulates BMDCs to polarize in different directions, RNA-Seq was performed on BMDCs treated with Mo-Pr or LPS for 4 h. This analysis revealed a total of 30,019 expressed genes, 29,223 of which were already known, and the remaining 796 genes were novel. There were 103,151 expressed transcripts, comprising 90,631 known transcripts and 12,520 novel transcripts. The PCA of the expression for the whole transcriptome showed that the treatment and control groups could be clearly distinguished, but the LPS and Mo-Pr groups had high similarity (Figure 8). In contrast, BMDCs treated with LPS or Mo-Pr for 48 h showed significant differences (Figure S1). Two outliers (LPS_1_1 and Mo_Pr_3_1) were removed using PCA, and further analysis was performed (Figure 8).

**Figure 9:**
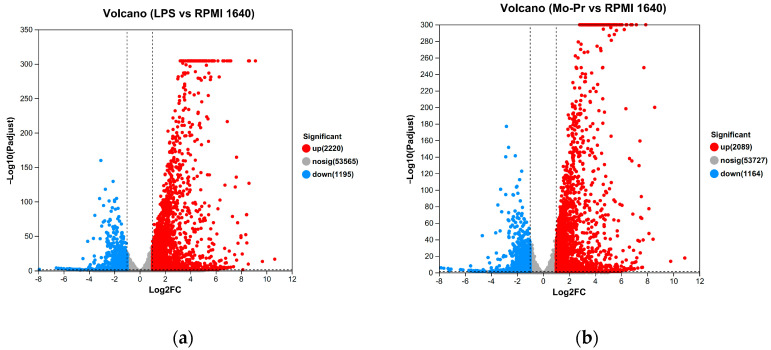
Volcano plot showing up- and downregulated mRNA transcripts in the LPS group or Mo-Pr group compared with the RPMI 1640 group: (**a**) LPS vs. RPMI 1640; (**b**) Mo-Pr vs. RPMI 1640.

**Figure 10:**
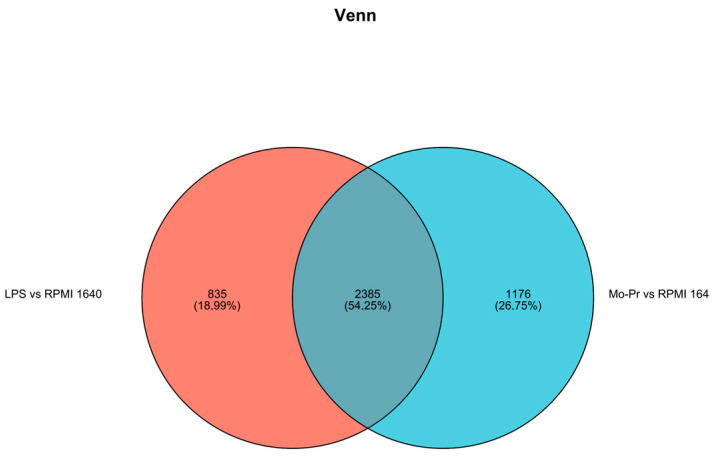
Venn diagram of DEGs.

Expression differences between the LPS and RPMI 1640 groups revealed that 3415 genes were differentially expressed, with 2220 genes upregulated and 1195 genes downregulated; similarly (Figure 9a), 3253 genes were differentially expressed between the Mo-Pr and RPMI groups, with 2089 genes upregulated and 1164 genes downregulated (Figure 9b). According to the Venn diagram of DEGs, there were 2385 shared DEGs between LPS- and Mo-Pr-treated BMDCs, accounting for 54.25% of the total DEGs, indicating the similarity of BMDCs treated with LPS or Mo-Pr (Figure 10).

**Figure 11:**
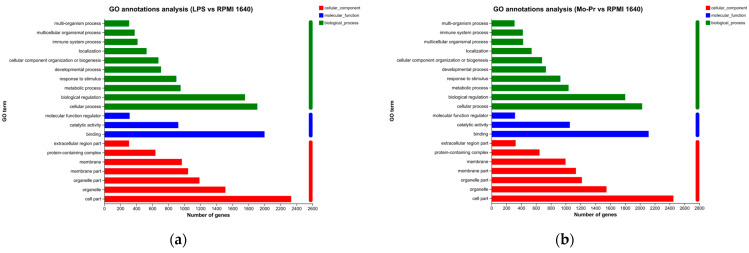
GO annotation analysis of DEGs: (**a**) LPS vs. RPMI 1640; (**b**) Mo-Pr vs. RPMI 1640.

To explore the functions of DCs regulated by LPS or Mo-Pr, the GO database was used to obtain the relevant functional information, and the importance of the function set for the differentially expressed genes was determined. Figure 11a,b show the number of genes and terms for the BP, CC, and MF categories from the GO enrichment analysis.

**Figure 12:**
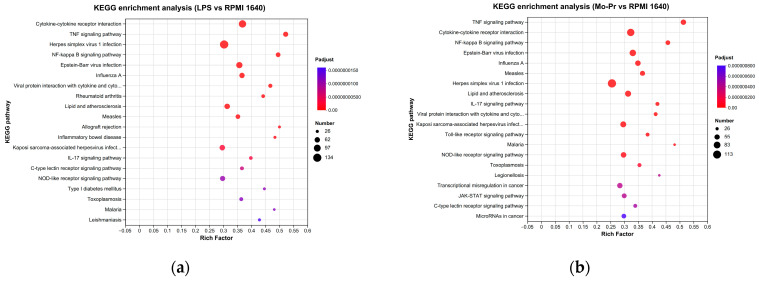
KEGG pathway enrichment analysis of DEGs: (**a**) LPS vs. RPMI 1640; (**b**) Mo-Pr vs. RPMI 1640.

To determine the pathways associated with DEGs, the number of DEGs in different KEGG pathways was tallied. The KEGG pathway enrichment analysis revealed a remarkable enrichment of DEGs in 330 canonical pathways in the LPS group and 329 canonical pathways in the Mo-Pr group. An enrichment bubble chart of the candidate genes provides a graphical representation of the top 20 most enriched pathways for DEGs. In the LPS group, the “cytokine receptor interaction” was the most enriched of the 20 pathways (Figure 12a), while the “TNF signaling pathway” was the most enriched in the Mo-Pr group (Figure 12b).

**Figure 13:**
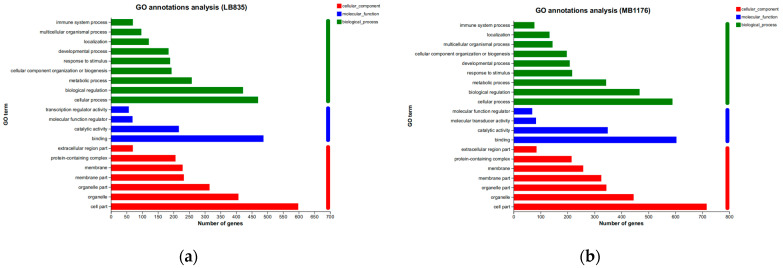
GO annotation analysis of two gene sets (LB835 and MB1176) with no overlap in the Venn diagram: (**a**) LB83; (**b**) MB1176.

**Figure 14:**
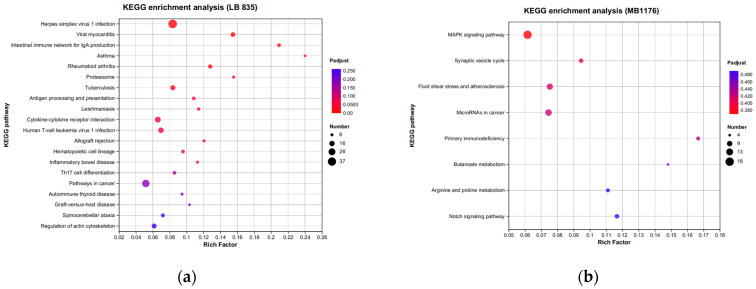
KEGG enrichment analysis of two gene sets (LB835 and MB1176) with no overlap in the Venn diagram: (**a**) LB83; (**b**) MB1176.

**Figure 15:**
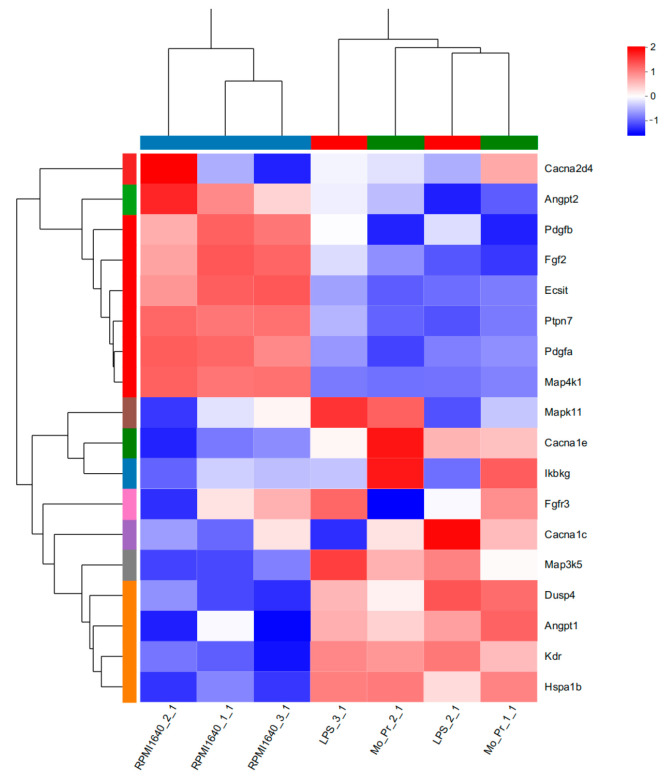
The 18 DEGs in which the MAPK signaling pathway was most enriched are listed in Table 2. Heatmaps of the 18 DEGs are shown.

**Figure 16:**
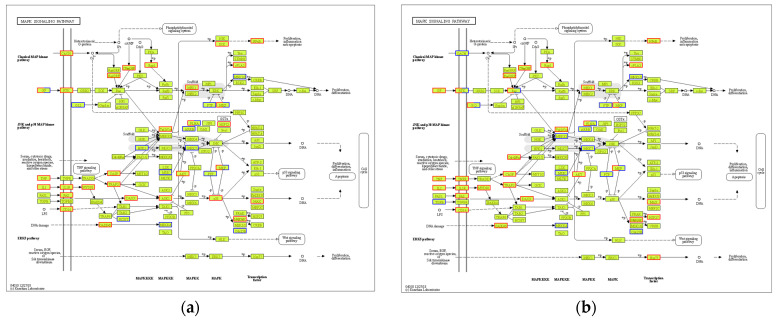
MAPK signaling pathway map of DEGs from the LPS group or the Mo-Pr group compared with the RPMI1640 group: (**a**) LPS vs. RPMI 1640; (**b**) Mo-Pr vs. RPMI 1640. The red boxes indicate up-regulated DEGs. The blue boxes indicate down-regulated DEGs.

KEGG analysis showed that BMDCs activated similar pathways 4 h after LPS or Mo-Pr treatment, and a similar result was obtained with PCA. However, flow cytometry and MLR experiments showed that Mo-Pr induced the polarization of BMDCs toward a Th2 phenotype, whereas LPS was generally considered to induce the polarization of BMDCs toward a Th1 phenotype. To clarify the difference between LPS and Mo-Pr treatment, GO annotation analysis (Figure 13a,b) and KEGG enrichment analysis (Figure 14a,b) were performed for two gene sets (LB835 and MB1176) that did not overlap in the Venn diagram. KEGG enrichment revealed that in the MB1176 group, the “MAPK signaling pathway” was the most enriched pathway. Therefore, we suggest that the MAPK signaling pathway may play an important role in the Mo-Pr-mediated activation of BMDCs toward Th2 polarization. The 18 DEGs for which the MAPK pathway was enriched are listed in Table 2 for reference. Heatmaps of the 18 DEGs are shown (Figure 15). A MAPK pathway map of DEGs from the LPS (Figure 16a) and Mo-Pr (Figure 16b) treatments compared to RPMI 1640 is shown.

**Figure:**
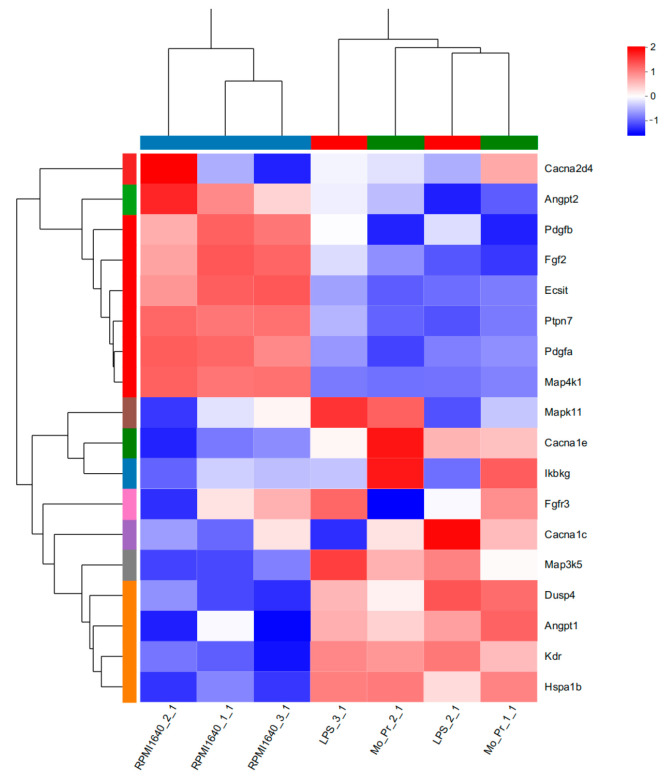


## 4. Discussion

*M. oleifera* is a nutritious alternative to staple foods that is gaining increasing attention. However, it should be noted that some individuals may be allergic to *M. oleifera* leaves. Previous studies have investigated *M. oleifera* leaf allergens [6,7,8], but the mechanism through which Mo-Pr or *M. oleifera* leaf allergen causes allergies as well as its effect on DCs remain unclear. Food allergy is a significant public health issue worldwide. The literature has extensively discussed the role of DCs in allergy. DC transplantation has demonstrated the importance of DCs in the generation of food-induced allergic reactions: in naive recipients without prior immunization, DCs from the spleen and Peyer’s patches of allergic mice induced the production of cow’s milk-specific IgG and IgE antibodies [26].

DC-induced Th2 cell differentiation requires DC maturation and polarization toward Th2. Mature DCs exhibit various characteristics, including decreased phagocytosis; an increased ability to stimulate T-cell differentiation; the increased secretion of cytokines; and increased levels of costimulatory factors, such as CD80/86 and MHC II, on their surface [13]. Our results confirmed the maturation of BMDCs from three aspects: an increase in costimulatory molecules, cytokine secretion, and the stimulation of lymphocyte proliferation. It is currently recognized that OX40L and TIM-4 play a crucial role in the polarization of DCs toward Th2 cells. Our study demonstrated that Mo-Pr-stimulated BMDCs upregulated the expression of OX40L and TIM-4 on the cell surface. This upregulation theoretically contributes to the differentiation of Th0 cells into Th2 cells, which was further confirmed in MLR experiments. In a separate study, OX40L was upregulated in an IL-33-dependent manner following intragastric immunization with peanut and CT [27]. Our experimental results also confirm this phenomenon, as shown in Figure 4a (The expression of IL-33 was recorded in the data of RNA-Seq). The expression of TIM-4 on DCs, another molecule characteristic of Th2 polarization, is also associated with Th2 differentiation. The binding of TIM-4 to T cells has been shown to affect the development of Th2 cells [17]. Treating patients with house dust mite (HDM) allergies using allergen-specific immunotherapy (SIT) that includes Der p 1 resulted in a decrease in TIM-4 expression in DCs and TIM-1 expression in T cells [28]. A study found that p300 and STAT6 induce TIM-4 expression in DCs [29]. After confirming the polarization of BMDCs, we investigated the cause of this polarization by performing an RNA-Seq analysis of BMDCs after 4 hours of stimulation. PCA analysis revealed that LPS and Mo-Pr had similar transcriptional profiles during the early stimulation of BMDCs. However, while the LPS stimulation of DCs is generally considered to be the Th1 type, our results indicate that the Mo-Pr stimulation of DCs is the Th2 type. We conducted an analysis of differential gene enrichment and hypothesized that the observed late change may be associated with the MAPK signal pathway.

Additionally, some allergens have been shown to act by binding to pattern recognition receptors (PRRs) that are capable of inducing Th2 responses. Ara h 1, a major peanut allergen, is reported to bind to the C-type lectin receptor (CLR) DC-specific intercellular adhesion molecule 3-grabbing nonintegrin (DC-SIGN) and stimulate human monocyte-DCs (MoDCs) to induce the Th2 differentiation of naive T cells in vitro [14]. No Th2-skewing effect was observed with deglycosylated Ara h 1, suggesting that the carbohydrate structure attached to allergens acts as an additional factor for the Th2-skewing effect [14]. Another research group tested the ability of different allergens to bind DC-SIGN on MoDCs and found that food allergens and respiratory allergens could also bind to this CLR [30]. Downstream of CLRs, the kinases ERK and Raf-1 are upregulated, and TNF-α, which is important for DC activation [31], is produced in a partially Raf-1-dependent manner [30]. Similar phenomena have been observed with other allergens. Glycans from HDM extracts induce Th2 differentiation by binding to the CLR dectin-2 on DCs and subsequently inducing cysteine leukotriene production [32]. The mannose receptor, another type of CLR, binds to and mediates the internalization of a variety of allergens such as Der p 1 and Der p 2 (HDM) and Ara h 1. Th treatment of BMDCs with mannose receptor RNAi reduced ovalbumin uptake and DC activation [33]. Toll-like receptor pathways are also thought to play a role in the allergen stimulation of DCs. It has been reported that the expression levels of TLR-2, TLR-7, and TLR-8 are significantly increased in OVA-treated BMDCs [33]. HDM could induce DC maturation dependent on TLR-2, TLR-4, and TLR-9 signaling [34]. According to another study, Der p 2, an analog of MD2 (the lipopolysaccharide-binding component of the Toll-like receptor (TLR) 4 signaling complex), binds LPS to Toll-like receptor 4 molecules when LPS signaling is extremely low [35]. By performing KEGG enrichment analysis, we found that in BMDCs treated with Mo-Pr for 4 h, differentially expressed genes exhibited the enrichment of the Toll-like receptor pathway, the C-type lectin receptor pathway, and the NOD-like receptor pathway. This suggests that potential allergens or adjuvants in Mo-Pr may increase the allergenicity of Mo-Pr by binding to these PRRs. Based on RNA-Seq data, Mo-Pr-treated BMDCs showed a slightly upregulated expression of Raf-1 (FC = 1.96116138664) and significantly upregulated expression of TLR-3 (FC = 29.0994367214), TLR-2 (FC = 29.0994367214), TLR-1 (FC = 3.30651539165), TLR-7 (FC = 3.07709026867), TLR-9 (FC = 2.37597650428), and TLR-6 (FC = 2.23650416544). Although these mRNAs were significantly upregulated, we could not conclude that Mo-Pr affects DC development through the same pathway as other genes were also altered. It is unclear whether this effect is a secondary or primary effect, which requires further investigation. Our future studies will continue to focus on these goals.

*M. oleifera* is a potential cash crop and a nutritious alternative to staple foods. In-depth research on *M. oleifera* allergy is helpful for the development and promotion of *M. oleifera* products. In this study, we demonstrated that Mo-Pr activated BMDCs and induced their differentiation into Th2-polarizing phenotypes and investigated the mechanism of *M. oleifera* allergy based on RNA-Seq. Based on these data, we will further investigate the mechanism of interaction between *M. oleifera* allergens and DCs.

## Figures and Tables

**Table 1:** Sequence of primers.

Primer	Sequence
Gapdh F	AAGAAGGTGGTGAAGCAGG
Gapdh R	GAAGGTGGAAGAGTGGGAGT
CCL17 F	TACCATGAGGTCACTTCAGATGC
CCL17 R	GCACTCTCGGCCTACATTGG
CCL22 F	AGGTCCCTATGGTGCCAATGT
CCL22 R	CGGCAGGATTTTGAGGTCCA

**Table 2:** The 18 differentially expressed genes in which the MAPK signaling pathway was most enriched.

Gene ID	Gene Name	Gene Description
ENSMUSG00000000489	Pdgfb	platelet-derived growth factor, B polypeptide (source: MGI Symbol; Acc: MGI: 97528)
ENSMUSG00000004110	Cacna1e	calcium channel, voltage-dependent, R type, alpha 1E subunit (source: MGI Symbol; Acc: MGI: 106217)
ENSMUSG00000004221	Ikbkg	inhibitor of kappa B kinase gamma (source: MGI Symbol; Acc: MGI: 1338074)
ENSMUSG00000022309	Angpt1	angiopoietin 1 (source: MGI Symbol; Acc: MGI: 108448)
ENSMUSG00000025856	Pdgfa	platelet-derived growth factor, alpha (source: MGI Symbol; Acc: MGI: 97527)
ENSMUSG00000031465	Angpt2	angiopoietin 2 (source: MGI Symbol; Acc: MGI: 1202890)
ENSMUSG00000031506	Ptpn7	protein tyrosine phosphatase, nonreceptor type 7 (source: MGI Symbol; Acc: MGI: 2156893)
ENSMUSG00000031530	Dusp4	Dual-specificity phosphatase 4 (source: MGI Symbol; Acc: MGI: 2442191)
ENSMUSG00000037225	Fgf2	fibroblast growth factor 2 (source: MGI Symbol; Acc: MGI: 95516)
ENSMUSG00000037337	Map4k1	mitogen-activated protein kinase kinase kinase kinase 1 (source: MGI Symbol; Acc: MGI: 1346882)
ENSMUSG00000041460	Cacna2d4	calcium channel, voltage-dependent, alpha 2/delta subunit 4 (source: MGI Symbol; Acc: MGI: 2442632)
ENSMUSG00000051331	Cacna1c	calcium channel, voltage-dependent, L type, alpha 1C subunit (source: MGI Symbol; Acc: MGI: 103013)
ENSMUSG00000053137	Mapk11	mitogen-activated protein kinase 11 (source: MGI Symbol; Acc: MGI: 1338024)
ENSMUSG00000054252	Fgfr3	fibroblast growth factor receptor 3 (source: MGI Symbol; Acc: MGI: 95524)
ENSMUSG00000062960	Kdr	kinase insert domain protein receptor (source: MGI Symbol; Acc: MGI: 96683)
ENSMUSG00000066839	Ecsit	ECSIT signaling integrator (source: MGI Symbol; Acc: MGI: 1349469)
ENSMUSG00000071369	Map3k5	mitogen-activated protein kinase kinase kinase 5 (source: MGI Symbol; Acc: MGI: 1346876)
ENSMUSG00000090877	Hspa1b	heat shock protein 1B (source: MGI Symbol; Acc: MGI: 99517)

## Data Availability

The data presented in this study are openly available in the NCBI database (BioProject: PRJNA1036700).

## Supplementary Materials

The following supporting information can be downloaded at: https://www.mdpi.com/article/10.3390/nu16010007/s1, Figure S1: Changes in the mRNA expression profile of BMDCs treated with LPS (1 μg/mL, 48 h) and Mo-Pr (50, 100, or 200 μg/mL, 48 h). Principal component analysis (PCA) of the whole transcriptome of BMDCs in the LPS group, Mo-Pr group, and RPMI 1640 group. Different colors in the figure represent different groups, and shapes represent different samples (*n* = 1).
