# Supplementary figures and images for: The Potential Role of Moringa oleifera Lam. Leaf Proteins in Moringa Allergy by Functionally Activating Murine Bone Marrow-Derived Dendritic Cells and Inducing Their Differentiation toward a Th2-Polarizing Phenotype
Source: Nutrients. 2023 Dec 19;16(1):7. doi: 10.3390/nu16010007 (PMC10780893; doi:10.3390/nu16010007)

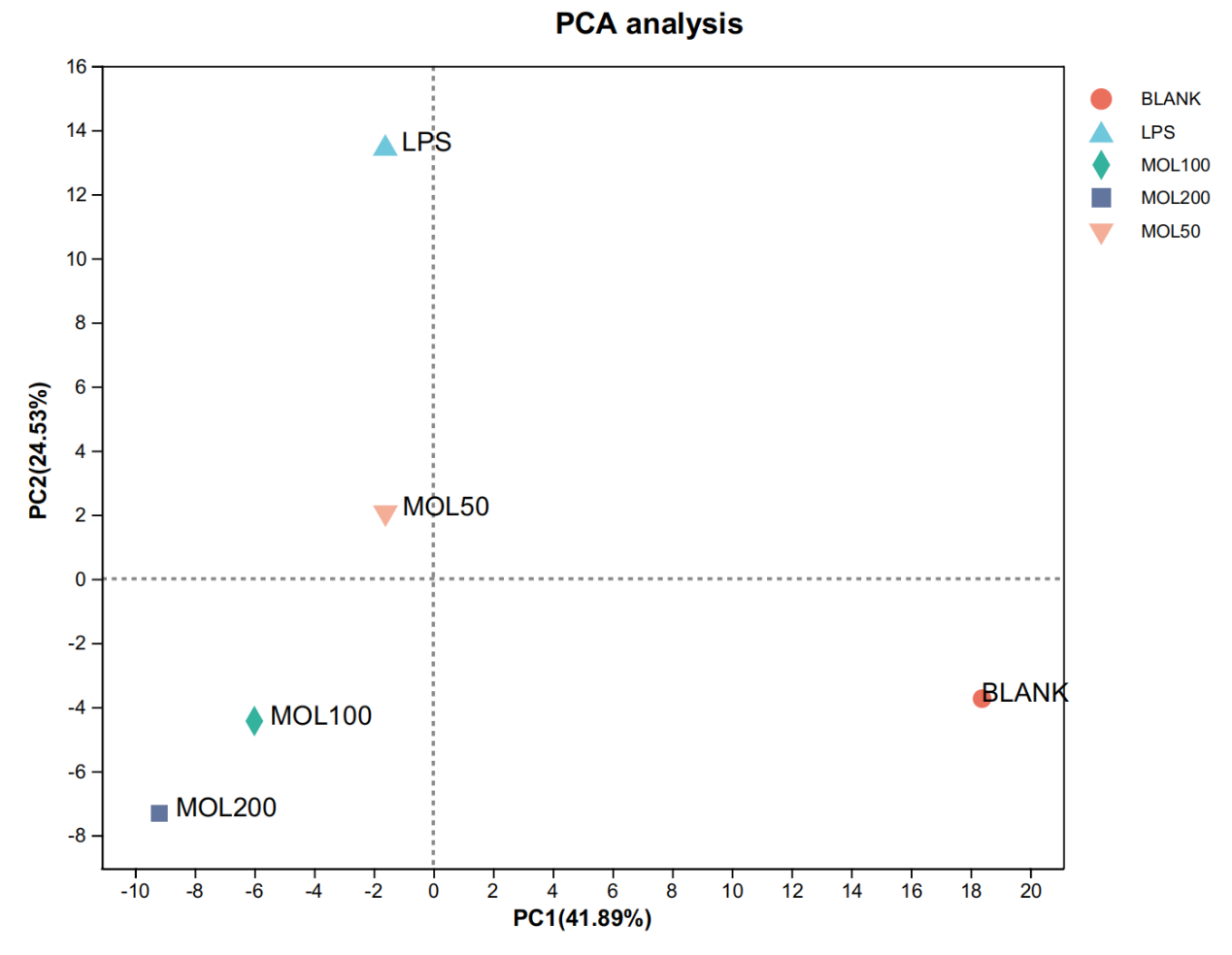

Supplement: Supplementary file 1 [file nutrients-16-00007-s001.zip › nutrients-2741345-supplementary.png]
